# The glycocalyx: a key target for treatment of severe acute pancreatitis-associated multiple organ dysfunction syndrome

**DOI:** 10.1007/s13577-025-01227-6

**Published:** 2025-05-24

**Authors:** Huijuan Li, Haiyun Wen, Jie Liu, Xinyu Luo, Boliang Pei, Peng Ge, Zhenxuan Sun, Jin Liu, Junjie Wang, Hailong Chen

**Affiliations:** 1 Department of General Surgery, The First Affiliated Hospital of Dalian Medical University, Dalian, 116011 Liaoning People’s Republic of China; 2 The First Affiliated Hospital of Dalian Medical University, Dalian, 116011 Liaoning People’s Republic of China; 3 Institute (College) of Integrative Medicine, Dalian Medical University, Dalian, 116011 Liaoning People’s Republic of China; 4 Laboratory of Integrative Medicine, The First Affiliated Hospital of Dalian Medical University, Dalian, 116011 Liaoning People’s Republic of China

**Keywords:** Glycocalyx, Severe acute pancreatitis, Multiple organ dysfunction syndrome, Endothelial dysfunction, Inflammation

## Abstract

The endothelial glycocalyx is a dynamic brush-like layer composed of proteoglycans and glycosaminoglycans, including heparan sulfate (HS) and hyaluronic acid (HA), and is an important regulator of vascular homeostasis. Its structure (thickness ranges from 20 to 6450 nm in different species) not only provides a charge-selective barrier but also serves to anchor mechanosensors such as the glypican-1 (GPC-1)/caveolin-1 (CAV-1) complex and buffers shear stress. In severe acute pancreatitis (SAP), inflammatory factors promote the expression of matrix metalloproteinases (MMPs) and heparinases, which degrade syndecan-1 (SDC-1) and HS, while oxidative stress disrupts HA-CD44 binding, leading to increased capillary leakage and neutrophil adhesion. This degradation process occurs before the onset of multiple organ dysfunction syndrome (MODS), highlighting the potential of the glycocalyx as an early biomarker. More importantly, the regeneration of glycocalyx through endothelial cell synthesis of glycosaminoglycans (GAGs) and shear stress-driven SDC recycling provides therapeutic prospects. This review redefines the pathophysiology of severe acute pancreatitis-associated multiple organ dysfunction (SAP-MODS) by exploring the glycocalyx’s central mechanistic role and proposes stabilizing glycocalyx structure as a potential strategy to prevent microcirculatory failure.

**Graphical Abstract:**

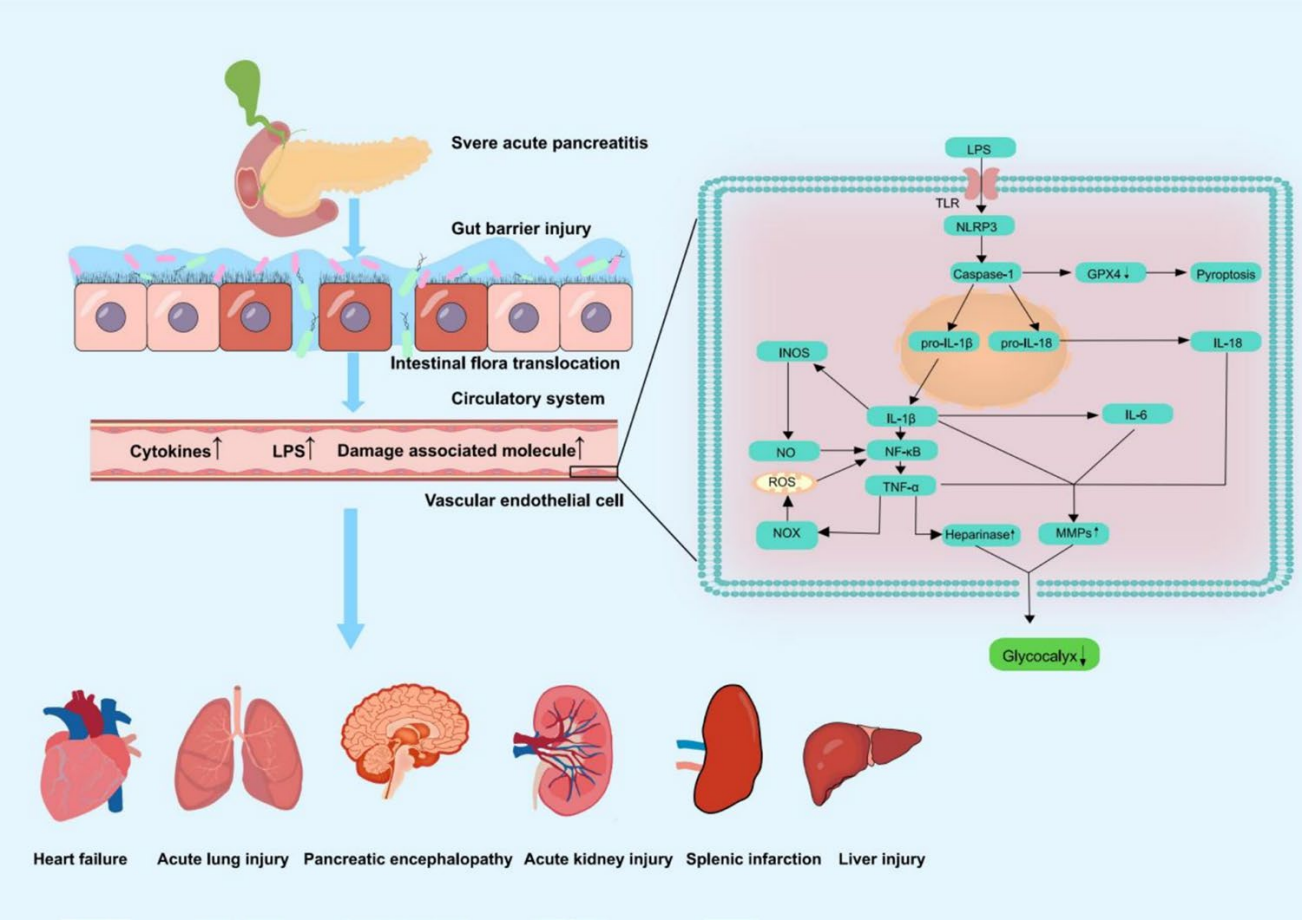

## Introduction

Acute pancreatitis is a common abdominal inflammatory condition. Mild cases do not cause organ failure or complications, while moderate to severe cases have organ failure or complications lasting less than 48 h. If organ failure persists beyond 48 h, the condition is classified as SAP [1]. SAP is often associated with distant organ injury, including the heart, lungs, brain, and kidneys [2, 3]. Approximately 20% of acute pancreatitis cases progress to SAP, which begins with systemic inflammatory response syndrome (SIRS), then MODS, and ultimately organ failure, with a mortality rate of 8–40% [4–6].

In the 1960s, Luft first used ruthenium red staining and electron microscopy to observe the glycocalyx structure [7]. The glycocalyx on the endothelial cell surface regulates cell adhesion, anticoagulation, vascular permeability, mechanoreception, and mechanotransduction [8, 9]. Studies indicate that the glycocalyx is critical in treating sepsis, malaria, traumatic brain injury and coronary atherosclerosis [10–13]. The glycocalyx is rich in HA, SDC-1, and HS; elevated plasma levels of these components indicate glycocalyx shedding [14–16]. Rats with pancreatitis exhibit elevated levels of reactive oxygen species (ROS) and MMPs, manifesting in glycocalyx and mitochondrial damage [17, 18]. Glycocalyx degradation increases capillary permeability, leading to capillary leak syndrome, the transition from SAP to MODS. Activation of pancreatic enzymes exacerbates necrosis, damages endothelial cells, and induces edema, hypoxia, and organ failure [19–21]. Recent studies have highlighted the pivotal role of the endothelial glycocalyx in systemic inflammatory diseases such as sepsis and atherosclerosis, where its degradation correlates with microcirculatory failure and organ dysfunction [8, 10, 13]. In acute pancreatitis, emerging evidence suggests that glycocalyx shedding precedes MODS onset, serving as both a biomarker and therapeutic target [22, 23]. This aligns with findings in sepsis models, where glycocalyx damage exacerbates vascular permeability and leukocyte adhesion [10, 24]. Our focus on glycocalyx in SAP-MODS stems from its potential to bridge pancreatic inflammation to systemic endothelial dysfunction, offering novel therapeutic avenues.

## Glycocalyx structure

**Fig. 1:**
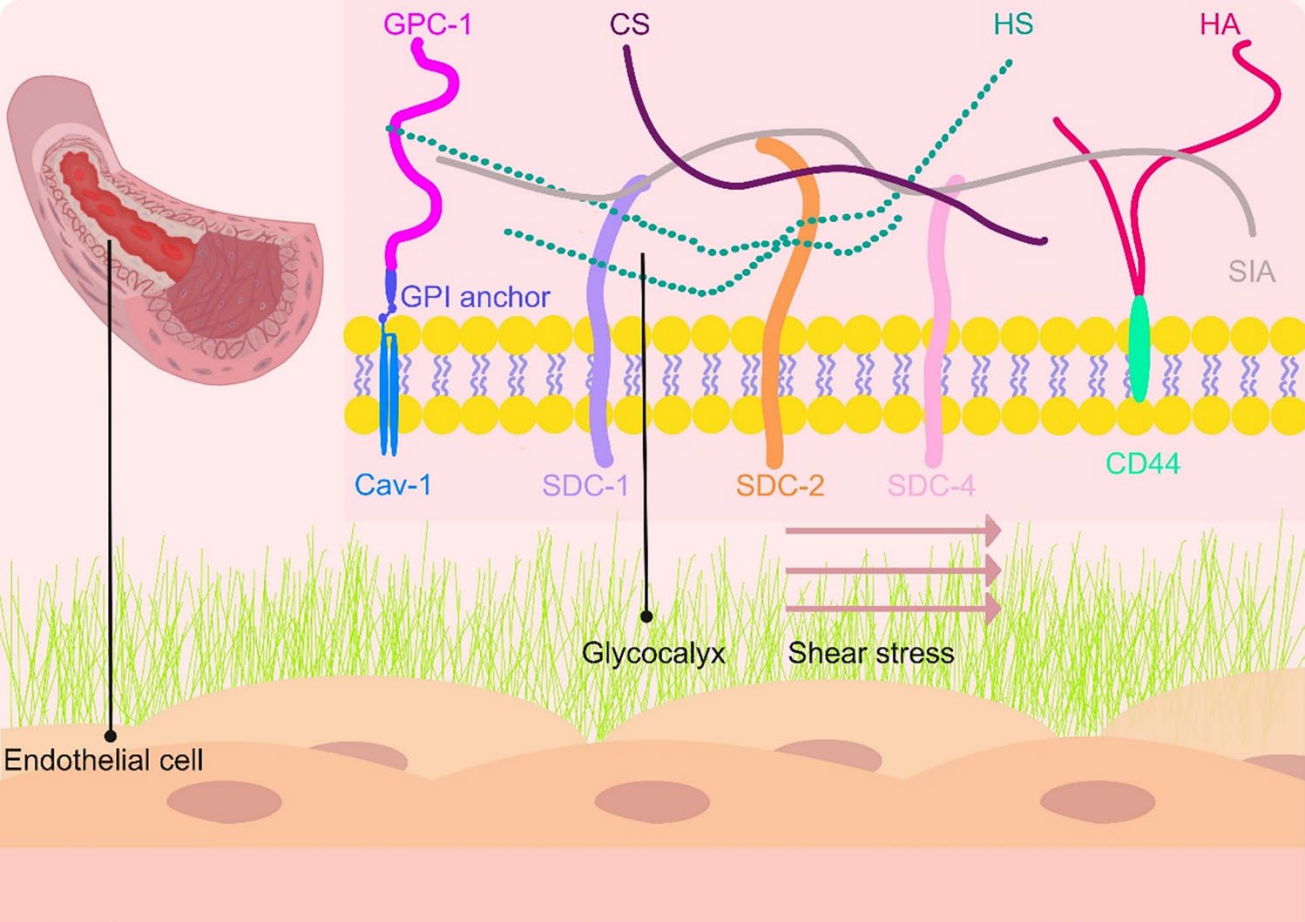
Schematic illustration of the Endothelial Glycocalyx Structure. The glycocalyx covers the luminal surface of endothelial cells and extends into the vessel lumen. This structure comprises intracellular, transmembrane, and extracellular segments and is primarily composed of proteoglycans and glycoproteins. Proteoglycans consist of core proteins linked to GAGs (e.g., SDC-1,SDC-2 and SDC-4) and SIA. The predominant components of the GAGs include HS, CS, and HA

The glycocalyx is a negatively charged brush-like structure that covers the surface of endothelial cells and extends into the vascular lumen (Fig. 1) [25, 26]. It is mainly composed of proteoglycans and glycoproteins [9]. Proteoglycans consist of core proteins with GAG chains and sialic acid (SIA) protein. The main components of the GAG are HS, representing 50–90%, HA and chondroitin sulfate (CS). The core protein is firmly incorporated into the cell membrane via either the transmembrane domain (syndecans) or glycosylphosphatidylinositol (GPI) anchor (glypicans) [27, 28]. The expression of the syndecan family on the vascular endothelium includes SDC-1, SDC-2 and SDC-4 [29]. The structure consists of an intracellular segment, a transmembrane domain, and an extracellular segment. The extracellular domain binds to the GAG. SDC-1 binds to HS and CS, while GPC-1 contains only an HS chain and lacks CS [26]. Unlike HS [30], GPC-1, an isoform of the GPC family, is expressed on the vascular endothelium and anchored to the plasma membrane's lipid rafts via C-terminal GPI. It plays a role in the mechanical conduction of blood flow shear force and can prevent endothelial cell dysfunction [9, 31]. Caveolae, a portion of lipid rafts, form through the integration of the protein CAV-1, which are upheld by the cytoskeleton. Recent studies highlight the role of GPC-1 in mechanotransduction via lipid raft-associated CAV-1, which integrates cytoskeletal dynamics to maintain endothelial integrity [32, 33]. Endothelial cell adhesion molecules are glycoproteins, mainly including selectin, integrin, and immunoglobulin families, which are used for cell recruitment and signal transduction. In addition, there are glycoproteins with coagulation, fibrinolytic, and hemostatic functions in the endothelial glycocalyx [34]. In addition, advanced glycocalyx fixation techniques (such as HPF and FS), along with visualization methods like TEM, reveal variations in glycocalyx thickness across vascular beds, providing new insights into its structural heterogeneity [35]. The thickness of glycocalyx varies across specimens (20 nm to 6.45 μm). (Table 1).

**Table 1:** Glycocalyx visualization

Setting	Immobilization before separation	Visualization	Fixative	Fixed time	Glycocalyx marker	Sample type	Glycocalyx thickness (nm)	Reference
In Vivo	Perfuse 2% lanthanum nitrate solution through the left ventricle until the kidney appears white	TEM	Lanthanum nitrate; Paraformaldehyde; Glutaraldehyde	24 h	Lanthanum nitrate	Mouse glomerular capillary	20–33^a^	[36]
	Perfused via the femoral vein with glutaraldehyde supplemented with cacodylate buffer + sucrose supplemented with lanthanum nitrate hexahydrate	TEM SEM FIB-SEM	Glutaraldehyde; Nitrate lanthanum Hexahydrate; Sucrose; Cacodylate buffer; Osmium tetroxide	2 h	Lanthanum nitrate	Rat pulmonary artery	1200^a^	[37]
		TEM	Glutaraldehyde; Alcian blue 8GX; MgCl2; Lanthanum nitrate; Glutaraldehyde; Sucrose	overnight	Lanthanum nitrate	Human skin capillary	200–700^a^	[38]
		µ-PIV	Non-fixed		Fluorescent polystyrene microspheres	Mouse cremaster muscle venules	520	[39]
In Vitro		TEM CLSM	HPF; FS; Uranyl acetate; Glutaraldehyde; Methanol; Water in acetone	48 h	WGA; DAPI; FITC CD44-IM7; CS-56	Human monocytic THP-1 cells	6450	[35]
		AFM	Non-fixed		Indentation measurements	HUVEC	123–231	[40]

*TEM* transmission electron microscopy, *SEM* scanning electron microscopy, *FIB*-*SEM* focus-ion-beam-scanning electron microscopy, *µ-PIV* microparticle image velocimetry, *CLSM* Confocal Laser Scanning Microscopy, *WGA* wheat germ agglutinin, *DAPI*, 4′6-diamidin-2-phenylindol, *FITC* fluorescein isothiocyanate conjugated, *CS*-*56* chondroitin sulfate antibody-56, *AFM* atomic force microscopy, *HPF* high-pressure freezing, *FS* osmium-free freeze substitution, *HUVEC* human umbilical vein endothelial cell

^a^The document did not provide specific data on the thickness of glycocalyx, but it was obtained with reference to the scale bar and relative to the cell dimensions

## Glycocalyx function

### Regulating vascular permeability

Notably, glycocalyx degradation exacerbates vascular permeability by disrupting VE-cadherin and occludin [41], while intact glycocalyx suppresses MMP-9-mediated inflammation [42, 43]. The glycocalyx at the apex of endothelial cells forms a permeability barrier. The intercellular junction serves as the primary channel through which water and other solutes traverse the vascular endothelium. Both the glycocalyx and junctional proteins resist solute entry, promoting endothelial cell connectivity [44]. When hemorrhagic shock occurs, the endothelial barrier is damaged, CS, HA, and HS in the glycocalyx are shed, and the permeability of pulmonary vessels is markedly elevated. Hydroxyethyl starch inhibits glycocalyx-degrading enzymes, facilitating the restoration of glycocalyx thickness and enhancing expression of VE-cadherin, occludin, and zonula occludens-1, reducing albumin–fluorescein isothiocyanate infiltration, and improving pulmonary vascular permeability [45]. In mice with lipopolysaccharide (LPS)-triggered acute respiratory distress syndrome, mitogen-activated protein kinase (MAPK) and nuclear factor kappa-B (NF-κB) signaling are activated, leading to up-regulation of matrix metalloproteinase-9 (MMP-9) and a reduction in SDC-1, indicating glycocalyx destruction. Inhibition of MMP-9 expression can maintain glycocalyx integrity and prevent increased vascular permeability [46].

### Mechanosensation and mechanotransduction

Mechanical sensors on endothelial cells detect shear stress and convert it into biochemical signals to trigger vascular adaptive response. Various glycocalyx factors, including SIA, HS, and SDC-1, are crucial in the signal transduction of blood flow shear force, affecting vascular function and related diseases. The glycocalyx is compromised in arteries with blood flow disorders, exacerbating oxidative stress and promoting atherosclerosis [47]. HS, isolated from endothelial cells under flow-conditioned, suppresses 5'-adenosine monophosphate-activated protein kinase signaling and enhances Angiopoietin-2 (Ang-2) protein expression in human primary lung microvascular endothelial cells. In humans and mice with sepsis, plasma HS levels peak earlier than Ang-2 levels, indicating a correlation between HS levels and organ damage in sepsis, as well as poor prognosis [48]. Le et al. showed that changes in SDC-1 mechanical tension generate a tension gradient in SDC-1 under fluid shear force, with SDC-1 interacting differently with the cytoskeleton depending on substrate stiffness, affecting integrin-mediated signaling and focal adhesion formation [49]. Therefore, SDC-1 adapts to mechanical stimuli by modulating its interactions with actin and signaling intermediates.

### Regulating leukocyte adhesion

Furthermore, SDC-1 dynamically regulates leukocyte adhesion through chemokine anchoring, offering a dual role in inflammation control [50, 51]. Leukocyte migration involves the leukocyte adhesion cascade and transendothelial migration into perivascular tissue. Leukocyte positioning depends on chemokines on the endothelial surface and cognate chemokine receptors on marginalized leukocytes. Furthermore, GAG in the glycocalyx is a vital substance that fixes chemokines on the endothelial surface [52]. Neutrophils, key immune cells, play a central role in the systemic inflammatory response, which is linked to glycocalyx degradation. In this process, neutrophil activation promotes adhesion to endothelial cells, consequently altering the structure of glycocalyx [53]. In vitro injury models in human umbilical vein endothelial cells demonstrated that tumor necrosis factor-alpha (TNF-α) treatment promotes neutrophil adhesion and reduces glycocalyx thickness. Treatment with heparinase III and hyaluronidase, enzymes degrading glycocalyx components, enhanced neutrophil adhesion. Interestingly, individual enzymes did not significantly affect neutrophil affinity for the endothelium [24].

## Endothelial dysfunction in SAP

Emerging evidence indicates that glycocalyx shedding precedes organ failure in SAP, serving as both a biomarker and a therapeutic target [22]. For instance, SAP-induced MMP-9 activation accelerates glycocalyx degradation, thereby directly linking pancreatic inflammation to systemic microcirculatory collapse [18]. The damage caused by SAP to the vascular endothelium is manifested as the release of inflammatory mediators and leukocyte adhesion, increased vascular permeability, changes in blood flow shear stress, microthrombosis, and microcirculatory disorders. It can eventually induce endothelial cell apoptosis and aggravate organ damage (Table 2).

**Table 2:** Endothelial dysfunction in SAP

Organ/tissue	Endothelial cell type	Damage mechanism	Glycocalyx function	Reference
Lung	PMVEC	Increase ICAM-1 and vascular permeability Promote vascular endothelial cell apoptosis	Regulate vascular permeability; Promote angiogenesis	[54, 55]
Kidney	RMEC	Elevated inflammatory factors and MMPs levels induce renal hypoperfusion and microcirculatory disturbances	Regulate vascular permeability; Prevent leukocytes and platelets from aggregating on blood vessels	[21, 56]
Brain	BMEC	Upregulated TNF-α and MMP-9; DecreasedZO-1 and claudin-5 increased BBB permeability	Reduce BBB permeability in rats with SAP	[17, 57]
Heart	CCEC	Promote inflammatory response and oxidative stress in cardiac tissue, leading to vascular congestion	Prevent leukocytes and platelets from aggregating on blood vessels; Reduce oxidative stress in vascular endothelial cells	[58, 59]
Intestine	ICE	Inflammation causes capillaries to leak	Regulate vascular permeability; Inhibit leukocyte adhesion	[60]
Liver	PVEC	Vascular obstruction causing portal hypertension	Regulate vascular permeability; Inhibit leukocyte adhesion	[61, 62]
Umbilical vein	HUVEC	Increased adhesion between platelets, leukocytes and vascular endothelium; Increased vascular permeability; Promotes microthrombosis	Reduce inflammatory response and high permeability of vascular endothelium	[19, 63, 64]

*PMVEC* pulmonary microvascular endothelial cell, *ICAM*-1 intercellular cell adhesion molecule-1, *RMEC* renal microvascular endothelial cell, *BMEC* brain microvascular endothelial cell, *BBB* blood–brain barrier, *NO* nitric oxide, *AEC* aortic endothelial cell, *vWF* von Willebrand factor, *sEPCR* soluble endothelial protein C receptor, *CCEC* cardiac capillary endothelial cell, *ICE* intestinal capillary endothelium, *PVEC* portal vein endothelial cell, *VEGF* vascular endothelial growth factor, *bFGF* basic fibroblast growth factor

## Role of glycocalyx in SAP-associated multiple organ dysfunction

The pathogenesis of SAP is complex and involves intracellular inflammatory pathways, enzyme activation, endotoxemia, and vascular endothelial damage (Table 3). In acute pancreatitis, early trypsin activation leads to autodigestion, causing extended inflammation and SIRS, which may progress to MODS [6, 65]. Glycocalyx degradation usually begins in the SIRS stage, when proinflammatory factors (TNF-α, IL-1β) activate endothelial cells and directly destroy the components of the glycocalyx (such as HS, HA) by activating MMPs [23]. Inflammatory responses increase oxidative stress and oxygen free radicals, which impair GAG synthesis. After glycocalyx degradation, the barrier function of endothelial cells is impaired, further aggravating systemic microcirculatory disorders and organ hypoperfusion [66–68]. This dysfunction affects multiple organs, with respiratory failure being the most common, and cardiovascular failure often leading to the worst outcomes [69].

**Table 3:** Compounds that degrade the glycocalyx

Degradation compound	Mechanism	References
Hyaluronidase	Degrades HA	[24]
Heparinase I/III	Degrades HS	[24, 70]
Histone deacetylase	Mediates the oxidative stress-induced upregulation of MMPs	[68]
MMPs	Cleaves proteoglycans	[46, 68, 71]
TNF-α	Activates MMPs and heparinases	[21, 24, 71, 72]
Interleukin	Promotes inflammation	[73–76]
STAT3	Promotes inflammation	[77]
Ricin toxin	Promotes inflammation	[78]

*STAT3* Signal transducer and activator of transcription 3

### SAP-associated myocardial dysfunction

Activation of trypsin during acute pancreatitis exacerbates pancreatic injury. Studies have shown that trypsin induces the release of calcium ions from vascular endothelial cells, causing coronary artery-dependent vasodilation and reducing the contractile sensitivity of smooth muscle [79]. Rats with acute pancreatitis had a 21% reduction in cardiac output, a 37% decrease in contractility, a 23% decline in diastolic tension, and a twofold increase in the cardiac tissue edema index [80].

ROS produced by acute pancreatitis stimulate NF-κB, leading to excessive release of cytokines such as interleukin-1β (IL-1β), interleukin-2 (IL-2), interleukin-18 (IL-18), interleukin-6 (IL-6), and TNF-α [81]. TNF-α activates nicotinamide adenine dinucleotide phosphate oxidase (NOX), producing ROS [2], creating a vicious cycle. TNF-α was significantly increased in the serum of SAP patients. TNF-α peaked on day 1 in patients with moderate/severe pancreatitis, whereas TNF-α peaked on day 4 in patients with mild pancreatitis. Therefore, TNF-α can be used as a marker of the severity of acute pancreatitis [82]. TNF-α activates heparanase and MMPs to degrade proteoglycans such as SDC-1 [71]. TNF-α damages cardiomyocyte mitochondria by inhibiting glycolysis and reducing mitochondrial adenosine triphosphate (ATP) production through promoting oxidative and nitrosative stress, which can result in compromised cardiac systolic function and unfavorable cardiac remodeling [83, 84]. IL-1β is a typical inflammatory mediator produced by nucleotide-binding domain, leucine-rich-containing family, pyrin domain-containing 3 (NLRP3) inflammasome, leading to myocardial hypertrophy, heart failure, and coronary atherosclerosis [85, 86]. Increased IL-1β expression and amylase activity in caerulein-triggered pancreatitis [87]. Myocardial ischemia can trigger NLRP3 inflammasome to activate caspase-1. The active caspase-1 converts pro-IL-18 and pro-IL-1β into IL-18 and IL-1β, which further induces an inflammatory response and triggers pyroptosis [74, 88]. NLRP3 inhibitor Oridonin can suppress the expression of IL-18 and IL-1β, alleviate inflammation, preserve myocardial function and improve myocardial remodeling by reducing NLRP3 expression [89]. IL-6 is also an inflammatory mediator and a key cytokine of the innate immune system [75]. Anti-viral cocktail therapy down-regulated the IL-6-STAT3-CXCL1/MCP-1 signaling pathway mediated by toll-like receptor 9 (TLR9), significantly reduced serum lipase and amylase, and decreased neutrophils and macrophages infiltration [90]. In cardiovascular diseases, elabela antagonizes angiotensin II-mediated negative changes in heart structure, heart dysfunction, and myocardial fibrosis by inhibiting the IL-6/STAT3/GPX4 pathway [91]. IL-6 is a trigger factor for systemic inflammation. Higher SDC-1 and thrombomodulin levels correlate with increased IL-6 levels [73]. Therefore, reduction of IL-6 and inflammatory cell accumulation may inhibit glycocalyx degradation during acute pancreatitis and protect the endothelium.

During the early stages of acute pancreatitis, the inflammatory response damages the intestinal barrier, allowing bacteria and endotoxins to enter the blood, triggering an inflammatory storm, sepsis, and even multiple organ failure [92]. Extensive research has highlighted the critical role of the gut-heart axis [93]. Gram-negative bacteria produce LPS, disrupting the intestinal barrier and causing dysbiosis [94, 95]. Studies have found a significant increase in LPS, which is rapidly detected by TLR4 receptors. This process triggers intracellular signaling cascades, leading to increased NLRP3 expression, promotion of myocardial fibrosis, and further acceleration of the progression of atrial fibrillation. Moreover, MCC950, an NLRP3 inflammasome inhibitor, has been found to effectively reduce atrial fibrosis. By suppressing NLRP3 expression, MCC950 attenuates the inflammatory response and mitigates the progression of atrial fibrillation [96]. This highlights the potential treatment role of targeting the NLRP3 pathway in managing cardiac complications associated with dysbiosis and LPS-induced inflammation.

During SAP, levels of endothelin-1 (ET-1) increase significantly [97]. ET-1, a vasoconstrictor peptide originating from endothelial cells, exerts its effects through endothelin receptors and is correlated with cardiovascular disease severity. The study indicated that after three weeks of ET-1 infusion in mice, both systolic and diastolic blood pressures increased by 10–12 mmHg compared to baseline [98]. Hypertension may cause extensive damage and shedding of the endothelial glycocalyx due to increased or irregular shear forces [99]. In addition, ET-1 can cause positive muscle strength enhancement, arrhythmia, myocardial hypertrophy and accelerate myocardial fibrosis through signal transduction in pathologic conditions such as chronic heart failure. Clinical studies have shown that elevated ET-1 levels after myocardial infarction are associated with poor prognosis [100].

Acute pancreatitis promotes the expression of NO and inducible nitric oxide synthase (iNOS), which activates the NF-κB signaling pathway, and mediates the increase induced by spinal cord cyclooxygenase-2 (COX-2) via IL-1β, and stimulates pain in the body [101]. NO activates guanylate cyclase and elevates cyclic Guanosine Monophosphate (cGMP). cGMP facilitates smooth muscle relaxation in blood vessels either by enhancing Ca^2+^ extrusion or by reducing the sensitivity of the myofilaments to Ca^2+^[102]. The increase in Ca^2+^ release also simultaneously induces endothelium-dependent vasodilation. NO is the primary factor involved in the late stage of vasodilation [79].

### SAP-associated lung dysfunction

Acute lung injury is the earliest and most common severe complication of SAP, with a mortality rate of about 60–70%. Its main clinical manifestations are refractory hypoxia and respiratory insufficiency. Pathologically, alveolar epithelial and pulmonary microvascular endothelial cells are damaged, and the permeability of microvascular basement membrane increases, leading to fluid accumulation in the interstitium or alveolar cavity [103]. SAP-associated lung injury is linked to oxidative stress and autophagy via the p62-Keap1-Nrf2 pathway. Nuclear factor erythroid 2-related factor 2 (Nrf2) knockout increases ROS production and autophagy in mouse BEAS-2B cells, exacerbating inflammation [104]. SIA within the glycocalyx regulates Nrf2 mediating signal transfer by fluid shear force in the vascular endothelium, thereby regulating the endothelium's redox state [47]. Phospholipase A2 is overactivated during SAP, destroying pulmonary surfactant and inducing inflammation through accumulation of eicosanoids and platelet-activating factor [105]. Pulmonary surfactant can minimize alveolar surface tension and prevent alveolar collapse and microatelectasis. Glycocalyx degradation can also lead to atelectasis. Injection of mice with heparinase I/III can reduce lung compliance, increase MMP-7 and MMP-9 expression, and cleave HS in the glycocalyx [70]. Wedelolactone inhibits caspase-1/11 activation and reduces IL-1β and gasdermin D levels, thereby alleviating pyroptosis, ferroptosis, and lung injury through glutathione peroxidase-4 (GPX4) mediation [106]. LPS injection in mice resulted in neutrophil infiltration, glycocalyx shedding, and increased capillary permeability. Plasma SDC-1 levels peaked at 24 h and returned to normal at 48 h, while lung glycocalyx structure recovered within 96 h [107]. These findings highlight the dynamic balance of glycocalyx degradation and restoration, suggesting that glycocalyx damage is a hallmark of SAP-associated lung dysfunction and a target for prevention and treatment.

### SAP-associated brain dysfunction

BBB (blood–brain barrier) disruption in patients with SAP may lead to secondary brain edema, which can further cause pancreatic encephalopathy [57]. In the acute pancreatitis group, 69% of the rats showed discontinuous glycocalyx covering the brain capillary endothelium, which led to increased capillary permeability, damaged basal lamina, and swollen glial endfeet [17]. MMP-9 is one of the elements contributing to the increase of BBB permeability. Elevated expression of MMP-9 in SAP [57, 68]. Therefore, reducing MMP-9 can inhibit glycocalyx degradation, protect the BBB and restore the function of cerebrovascular endothelium. Inflammatory factors stimulate increased brain microvascular permeability, such as IL-1, TNF-α, and IL-6. TNF-α disrupts the BBB by inducing C-X-C motif chemokine ligand 10 (CXCL10) secretion and promoting peripheral T lymphocyte infiltration. TNF-α-induced necroptosis causes damage to cerebral endothelial cells in blood vessels, neuroinflammation, and dysregulation of astrocyte Kir4.1 [108, 109]. In the traumatic brain injury model, SDC-1 in rat cerebral tissue decreased while increased in serum. This was mediated by the activation of the S100B/RAGE signaling pathway [110]. Glycocalyx degradation after cardiac arrest and resuscitation in rats induces vascular endothelial adhesion factor (VCAM-1), intercellular cell adhesion molecule-1 (ICAM-1), iNOS, and COX-2 expression, exacerbating neuronal loss, glial activation, and brain edema. Hydrocortisone can restore glycocalyx and improve the 7-day survival rate after brain injury [111]. Therefore, the glycocalyx may serve as a therapeutic target to restore BBB function.

### SAP-associated renal dysfunction

Among patients with acute pancreatitis, the occurrence rate of acute kidney injury (AKI) is 7.9%, with a mortality rate of 1.4%. In the AKI subgroup, the mortality rate was 8.8%, while in the non-AKI subgroup, the mortality rate was 0.7% [112]. SAP triggers the release of a multitude of inflammatory factors, including the caspase-1-dependent liberation of IL-18 and IL-1β. These inflammatory reactions are associated with NLRP3 inflammasome activation in macrophages [113]. Activation of the NF-κB pathway further promotes NLRP3 transcription and inflammatory cytokines such as TNF-α and IL-6, impairing renal vascular cells [76, 114]. TNF-α began to rise 3 h after the onset of SAP, peaking at 6 h. Renal blood perfusion decreased significantly by 6 h and reached its lowest point at 24 h, with glycocalyx thinning becoming evident [21]. A clinical study showed that children with sepsis had glycocalyx loss after unbalanced crystalloid resuscitation. Angiotensin II, annexin A5, and SDC-1 in the blood peaked after 6 h and were associated with increased risk of metabolic acidosis and AKI. Glycocalyx recovery in patients takes approximately 24 h after crystalloid resuscitation, but may take up to 5 days in animal models [115]. The degradation and recovery of the glycocalyx appear to follow a dynamic equilibrium. Changes in inflammatory factors and plasma osmolality can shift this equilibrium. Even when the same organ is affected, the recovery time varies between organisms. The commonality is that vascular damage can be mitigated when the factors contributing to glycocalyx degradation are restored to baseline or brought under control.

### SAP-associated other organs dysfunction

Patients with pancreatitis and complex vascular problems may experience splenic infarction. This may be caused by an inflammatory response in the tissue surrounding the spleen's blood vessels, which can exert pressure on the blood vessels and lead to splenic infarction [116]. The liver, as a neighboring organ of the pancreas, is often affected by collateral damage during SAP. The JAK2/STAT3 pathway plays a key role in regulating the inflammatory response in SAP. The expression levels of phosphorylated Janus kinase 2 (pJAK2) and phosphorylated signal transducer and activator of transcription 3 (pSTAT3) are elevated in liver tissue [117]. A recent study showed that a glycocalyx-mimetic nanoparticle library may facilitate drug delivery [118]. For patients with SAP- associated liver damage, glycocalyx-mimetic nanoparticles are expected to transport drugs and target them to the liver while controlling inflammation.

## Glycocalyx targeted therapy for MODS

### Key protective molecules of the glycocalyx

In basic scientific studies, various substances have been demonstrated protective effects on the glycocalyx (Table 4). Hydrocortisone and dexamethasone prevent TNF-α-triggered sloughing of the glycocalyx, thereby improving vascular endothelial function [21, 119, 120]. Phillyrin has also been shown to reduce glycocalyx shedding by inhibiting LPS-induced cathepsin L and ROS production. It further suppresses the NF-κB and MAPK signaling pathways, leading to decreased inflammatory cytokine levels [76]. Furthermore, Fraxin, Poria cocos and albumin-bound sphingosine-1-phosphate can reduce MMP levels, thereby alleviating glycocalyx damage. Poria cocos, in particular, has shown efficacy in improving AKI caused by increased fluid accumulation in SAP [18, 46, 121]. Recombinant syndecan-1 (rSyn-1) integrates into the plasma membrane through its lipophilic transmembrane domain and subsequently interacts with the actin cytoskeleton via its linker proteins to promote glycocalyx regeneration. Following rSyn-1 treatment, significant improvements were observed in the nanomechanical properties of both the endothelial glycocalyx and the cortical cytoskeleton [40]. Moreover, although Ang-1 and Ang-2 are not known drugs for protecting the glycocalyx, intervening in Ang-1 and Ang-2 may serve as targets for inhibiting glycocalyx degradation. Ang-1 treatment can improve cardiac diastolic capability in mice [122]. Anti-Ang2 antibody can effectively protect endothelial glycocalyx function and improve cardiovascular remodeling after myocardial ischemia [123]. In addition, Ang-1 mimetic and the inhibition of circulating Ang-2 could reduce vascular permeability [78]. Similarly, Secreted Protein Acidic and Rich in Cysteine regulates leukocyte recruitment, reduces capillary leakage, and restores glycocalyx integrity [72]. Colivelin, a synthetic form of the mitochondrial peptide humanin, can suppress STAT3 activation, thereby improving glycocalyx structure and safeguarding endothelial function [77]. Mesenchymal stem cell therapy can reduce serum amylase and TNF-α concentrations, increase serum IL-10 concentrations, reduce brain microvascular endothelial cell apoptosis, up-regulate claudin-5 levels, and reduce MMP-9 levels [57, 68]. In the rat acute lung injury model, Tanshinone IIA inhibited inflammation and oxidative stress, reduced glycocalyx degradation, and improved lung function [124].

**Table 4:** Key protective molecules of the glycocalyx

Protective compound	Mechanism	References
Hydrocortisone	Inhibits inflammation triggered by TNF-α	[111, 119, 120]
Phillyrin	Phillyrin inhibits NF-κB and MAPK activation, reducing inflammatory factors and glycocalyx degradation	[76]
sphingosine-1-phosphate	Reduces MMP-9 expression	[121]
Poria cocos	Reduces MMP-9 expression	[18]
Fraxin	Reduces MMP-9 expression	[46]
rSyn-1	Integrates into the membrane and links to the actin cytoskeleton to promote glycocalyx regeneration	[40]
Anti-Angpt2 antibody	Inhibits Angpt2-mediated heparanase expression	[123]
Ang-1	Diminishes vascular permeability and enhances myocardial diastolic function	[122]
Colivelin	Inhibits inflammation by inhibiting STAT3 signaling	[77]
Secreted Protein Acidic and Rich in Cysteine	Regulates the recruitment of leukocytes, diminishes capillary leakiness, and reinstates the integrity of the glycocalyx	[72]
Tanshinone IIA	Tanshinone IIA via nanoemulsion reduced MMP-9 levels and glycocalyx degradation in the lungs	[124]

### Clinical drugs with glycocalyx protective effects

In clinical studies, several marketed drugs have been confirmed to have protective effects on the glycocalyx (Table 5). Unlike sepsis, where treatments are primarily directed against LPS-induced inflammatory responses (e.g., anti-endotoxin antibodies), SAP-induced glycocalyx damage is predominantly driven by pancreatic enzyme activation (e.g., trypsin) and intestinal endotoxin release. Combined inhibition of trypsin (ulinastatin) and glycocalyx degradation (sulodexide) may have therapeutic advantages against SAP, addressing both enzymatic and inflammatory damage [18, 125, 126]. In a multicenter clinical study, both dexamethasone and albumin administration were found to have protective effects on vascular endothelial glycocalyx in patients undergoing abdominal surgery [127]. Sevoflurane offers protection to the glycocalyx, with patients in the sevoflurane group exhibiting significantly reduced concentrations of glycocalyx markers in the blood (such as SDC-1, HS, and HA) and enzymes responsible for glycocalyx degradation (such as MMP-9 and cathepsin-B), compared to the control drug propofol [128]. Sulodexide, a type of GAG, with heparin sulfate accounting for 80% and dermatan sulfate accounting for 20%, exhibits anti-thrombosis characteristics, promotes fibrinolysis, and can induce vasodilation through endothelium-dependent NO [102, 129]. Furthermore, ulinastatin is a serine protease inhibitor that inhibits trypsin and is clinically used to treat pancreatitis, disseminated intravascular coagulation, shock, and sepsis [130]. It can protect the integrity of the pulmonary endothelial glycocalyx and inhibit heparanase activity [126]. Finally, early injection of tranexamic acid before admission and during hospitalization can reduce plasma syndecan-1 levels, alleviate endothelial glycocalyx damage, and enhance vascular repair mechanisms [131, 132].

**Table 5:** Clinical drugs with glycocalyx protective effects

Clinical drug	Mechanism	References
Dexamethasone	Inhibits inflammation triggered by TNF-α	[21]
Sevoflurane	Reduces MMP-9 expression	[128]
Sulodexide	Reduces GAG metabolism and enhances abundance of precursors for endothelial GAGs	[102, 129]
Ulinastatin	Inhibits heparanase activity	[126]
Tranexamic acid	Reduces fibrinolysis and inhibits the activities of A Disintegrin and Metalloproteinase-17, TNF-α, and MMPs	[131, 132]

In clinical settings, there is relatively less research on drugs aimed at protecting the glycocalyx, with more conclusions drawn from animal experiments. However, this also provides us with some inspiration, suggesting that potential therapeutic targets may be the breakthrough point for future clinical drug development.

## Summary

Glycocalyx degradation in SAP is not limited to a single organ but represents a systemic vascular endothelial insult, resulting from widespread inflammatory and enzymatic activation. As a key structural component of the endothelial barrier, glycocalyx integrity is vital for maintaining microvascular homeostasis across multiple organs [22, 60]. The observed injuries in the lungs, heart, kidneys, and brain share a common mechanistic hallmark: glycocalyx shedding, increased vascular permeability, neutrophil adhesion, and oxidative stress [5, 17, 21, 104]. Therefore, glycocalyx damage not only manifests as local pathologic changes in the organ but is also likely to be one of the common pathologic mechanisms for the progression of SAP to MODS.

SAP usually involves multiple organs, and its pathogenesis is associated with various factors, such as pancreatic enzyme activation, proinflammatory factors, and endotoxin release. The synergistic effects of multiple signaling pathways can lead to systemic microcirculatory disorders, triggering various organ dysfunctions. The glycocalyx plays a key role in maintaining microvascular homeostasis by regulating hemodynamics, vascular permeability, and cell adhesion. Although the protective role of glycocalyx in SAP-associated MODS has been explored, current studies mainly focus on the acute phase, with limited understanding of the glycocalyx repair mechanisms and long-term organ recovery [4, 133]. Research on glycocalyx protectants (such as phillyrin and sphingosine-1-phosphate) is still in its early stages and relies on animal models and in vitro experiments [76, 121]. There is insufficient clinical trial data to confirm their efficacy and safety in humans. Future studies should explore glycocalyx regeneration and its role in SAP-associated MODS to achieve better clinical outcomes.

## Data Availability

The datasets used and/or analysed during the current study are available from the corresponding author on reasonable request.

## Abbreviations

HS: Heparan sulfate
HA: Hyaluronic acid
GPC-1: Glypican-1
CAV-1: Caveolin-1
SAP: Severe acute pancreatitis
MMPs: Matrix metalloproteinases
SDC-1: Syndecan-1
MODS: Multiple organ dysfunction syndrome
GAGs: Glycosaminoglycans
SAP-MODS: Severe acute pancreatitis-associated multiple organ dysfunction
SIRS: Systemic inflammatory response syndrome
TNF-α: Tumor necrosis factor-alpha
SIA: Sialic acid
CS: Chondroitin sulfate
GPI: Glycosylphosphatidylinositol
HPF: High pressure freezing
FS: Freeze substitution
TEM: Transmission electron microscopy
SEM: Scanning electron microscopy
FIB-SEM: Focus-ion-beam- scanning electron microscopy
µ-PIV: Microparticle image velocimetry
CLSM: Confocal laser scanning microscopy
WGA: Wheat germ agglutinin
DAPI: 4′,6-Diamidin-2-phenylindol
FITC: Fluorescein isothiocyanate conjugated
CS-56: Chondroitin sulfate antibody-56
AFM: Atomic force microscopy
HUVEC: Human umbilical vein endothelial cell
LPS: Lipopolysaccharide
MAPK: Mitogen-activated protein kinase
NF-κB: Nuclear factor kappa-B
MMP-9: Matrix metalloproteinase-9
Ang-2: Angiopoietin-2
PMVEC: Pulmonary microvascular endothelial cell
ICAM-1: Intercellular adhesion molecule-1
RMEC: Renal microvascular endothelial cell
BMEC: Brain microvascular endothelial cell
BBB: Blood–brain barrier
NO: Nitric oxide
AEC: Aortic endothelial cell
vWF: Von willebrand factor
sEPCR: Soluble endothelial protein C receptor
CCEC: Cardiac capillary endothelial cell
ICE: Intestinal capillary endothelium
PVEC: Portal vein endothelial cell
VEGF: Vascular endothelial growth factor
bFGF: Basic fibroblast growth factor
STAT3: Signal transducer and activator of transcription 3
ROS: Reactive oxygen species
IL-1β: Interleukin-1β
IL-2: Interleukin-2
IL-18: Interleukin-18
IL-6: Interleukin-6
NOX: Nicotinamide adenine dinucleotide phosphate oxidase
ATP: Adenosine triphosphate
NLRP3: Nucleotide-binding domain, leucine-rich-containing family, pyrin domain-containing 3
MCP-1: Monocyte chemotactic protein-1
TLR9: Toll-like receptor 9
ET-1: Endothelin-1
iNOS: Inducible nitric oxide synthase
COX-2: Cyclooxygenase-2
cGMP: Cyclic guanosine monophosphate
Nrf2: Nuclear factor erythroid 2-related factor 2
GPX4: Glutathione peroxidase-4
CXCL10: C-X-C motif chemokine ligand 10
RAGE: Receptors for advance glycation endproducts
VCAM-1: Vascular endothelial adhesion factor
ICAM-1: Intercellular cell adhesion molecule-1
AKI: Acute kidney injury
pJAK2: Phosphorylated janus kinase 2
pSTAT3: Phosphorylated signal transducer and activator of transcription 3
rSyn-1: Recombinant full-length syndecan-1
